# Associations of Diet with Urinary Trimethylamine-N-Oxide (TMAO) and Its Precursors among Free-Living 10-Year-Old Children: Data from SMBCS

**DOI:** 10.3390/nu14163419

**Published:** 2022-08-19

**Authors:** Yiming Dai, Jiming Zhang, Zheng Wang, Sinan Xu, Qinyu Zhang, Zhiping Duan, Ruonan Tan, Xiaojuan Qi, Jianqiu Guo, Xiuli Chang, Chunhua Wu, Zhijun Zhou

**Affiliations:** 1 Key Laboratory of Public Health Safety of Ministry of Education, Key Laboratory of Health Technology Assessment of National Health Commission, School of Public Health, Fudan University, No.130 Dong’an Road, Shanghai 200032, China; 2 Zhejiang Provincial Center for Disease Control and Prevention, No.3399 Binsheng Road, Hangzhou 310051, China

**Keywords:** trimethylamine-N-oxide, diet, dietary diversity, 24 h dietary recall, school-age children

## Abstract

Trimethylamine-N-oxide (TMAO), a diet-derived cometabolite linked to cardiometabolic disease, has been associated with elevated dietary status, particularly in people with kidney failure and adults with dietary modulations. However, the influence of the current diet on TMAO levels in free-living children has not been adequately described. This study was to explore associations of food compositions and dietary diversity with urinary TMAO and its precursor concentrations. Urinary TMAO and its precursor concentrations of 474 healthy children from the Sheyang Mini Birth Cohort were quantified by ultra-performance liquid chromatography–Q Exactive high-resolution mass spectrometer (UPLC-Q Exactive HRMS). Individual food compositions from 24 h dietary recall data were classified into 20 groups and diversity scores were calculated according to the guidelines of the Food and Agriculture Organization of the United Nations (FAO). Associations of urinary TMAO and its precursors with food compositions and dietary diversity scores were assessed by generalized linear regression models. In models adjusted for potential confounders, urinary TMAO was significantly associated with intakes of fish (β, regression coefficient = 0.155, *p* < 0.05) and vegetables (β = 0.120, *p* < 0.05). Eggs intake showed positive associations with TMAO’s precursors (trimethylamine: β = 0.179, *p* < 0.05; choline: β = 0.181, *p* < 0.05). No association between meat intake and TMAO was observed, whereas meat and poultry intakes were related to the levels of acetyl-L-carnitine and L-carnitine (β: 0.134 to 0.293, *p* < 0.05). The indicators of dietary diversity were positively correlated to TMAO concentration (β: 0.027 to 0.091, *p* < 0.05). In this free-living children-based study, dietary factors were related to urinary TMAO and its precursors, especially fish, meat, and eggs. As such, dietary diversity was positively related to the level of TMAO.

## 1. Introduction

Trimethylamine-N-oxide (TMAO), a methylamine osmolyte, has gained attention due to being related to risks of major adverse cardiovascular events [1,2,3], kidney injury [4], stroke recurrence [5,6], and Alzheimer’s disease [7] among adults in epidemiological studies. Meanwhile, TMAO has been shown to directly cause atherosclerosis and thrombosis [8,9], promote platelet aggregation [10], and induce vascular inflammation and endothelial dysfunction [11]. Moreover, a study of 115 children and adolescents with pediatric chronic kidney disease found that methylamines were related to blood pressure abnormalities and chronic kidney risk [12]. A case-control study of metabolomics illustrated that children with autism spectrum disorders (ASD) had higher concentrations of TMAO in plasma [13]. TMAO concentrations depend partly on diet and lifestyle choice [14,15,16] and, therefore, have been proposed as a target for dietary modulation and behavioral interventions aimed at preventing the hazard of TMAO [17,18,19]. Childhood is an important period of developing dietary habits and lifestyles [20]. Thus, it is valuable to identify dietary determines that contributed to the urinary TMAO concentration among children.

In addition, TMAO is a gut microbiota-related metabolite, and changes to human gut microbiota and micro metabolites can occur after dietary modifications [18]. Intakes of animal foods such as red meat as well as plant foods have been found to modify the production of TMAO [21]. The intestinal microbiota metabolizes nutrient precursors of TMAO, such as choline and L-carnitine, which are abundant in animal foods, to produce trimethylamine (TMA). TMA is further metabolized to TMAO by the flavin-containing enzyme monooxygenase 3 (FMO3) in the liver [22,23]. In addition, TMAO also originates from direct consumption of TMAO or TMA-rich products [16].

Hence, the formation of TMAO is governed by a fragile balance among diet, gut bacteria, and host physiology [24]. Previous studies have demonstrated the correlations between fish intake and urinary and plasma TMAO [25,26]. For other certain dietary factors (such as red meat and eggs), there is extensive conflicting literature [27,28]. For instance, several studies found egg consumption was positively related to TMAO [29,30], but some did not [31,32]. Meat products and dairy food also revealed the same conclusions in human adults [14,33]. Furthermore, most of the studies were conducted on chronic kidney disease (CKD) patients with dietary intervention [12,34]. However, few studies systematically evaluated the relations between all dietary components and urinary TMAO concentrations in school-age children. Except for diet, factors influencing urinary TMAO are different and complex, including the kidney function, the activity of liver FMO3 [22], gut microbiome composition [9], and demographics (age, sex) [35]. Due to the variety and complexity of factors, it is vital to establish which dietary component significantly determined the increase in urinary TMAO concentrations in free-living children.

In the present study, we utilized a simple, high throughput method for the analysis of urinary TMAO and precursors and dietary data from 24 h recalls to explore the associations of all dietary components with TMAO and five precursors in a prospective birth cohort study of free-living school-age children.

## 2. Methods

### 2.1. Participants

This study was embedded in the Sheyang Mini Birth Cohort Study (SMBCS), a prospective longitudinal birth cohort, which enrolled 1303 pregnant women in Sheyang County, Jiangsu Province, China from June 2009 through January 2010 [36]. A total of 499 10-year-old children were followed-up at Sheyang Maternal and Child Care Center in August 2019. The 10-year-old children accompanied by their caregivers were willing to complete a questionnaire survey, urine collection, and physical examination. Fifteen children were excluded due to lack of social-demographic information (*n* = 7) and urine TMAO concentrations (*n* = 8). Ten children with daily abnormal energy intakes based on 24 h dietary survey were also excluded. The final sample size was 474. All participants signed the informed consent forms. Ethics approval (IRB#2021-02-0875) was obtained by the Ethics Committees of School of Public Health, Fudan University.

### 2.2. Dietary Assessment

Food intake data were collected using the 24 h dietary recall method. Children were face-to-face asked about what had eaten in the past 24 h by a trained investigator with the help of their caregivers and aimed to collect information on detailed food consumption. All the food was divided into 20 food groups according to Chinese Food Composition Tables (CFCT) 2004 and 2009 (National Institute of Nutrition and Food Safety, China CDC). The weight of food composition was added up based on the consumption of 20 food groups. The frequency of eating different kinds of fish and aquatic products was investigated using a simple food frequency questionnaire (FFQ). Respondents were asked how often they consumed the specified kind of each fish or aquatic products during the recent 3 months. Three possible frequency categories ranged from never to >3 times/week. Lastly, children’s dietary diversity was evaluated using dietary diversity score (DDS) and DDS_10_ according to the guidelines of the Food and Agriculture Organization of the United Nations [37], as shown in Table S1. Briefly, DDS was calculated based on the sum of the total number of food groups without amount requirement, while DDS_10_ was calculated with a minimum intake amount requirement. The child received one point if they consumed at least 10 g from a single food group, except that the threshold for fats and oils was 2 g. Other food groups were not included in the calculation of DDS_10_. By adding up the scores of different food groups, the DDS_10_ score ranged from 1 to 9. Food variety score (FVS) was on behalf of the categories of food children consumed in the past 24 h.

### 2.3. Analysis of TMAO and Precursors in Urine

Urine samples were pretreated and detected by a simple dilute and shoot method modified from previous research [38]. Briefly, an aliquot of a 100 μL urine sample was transferred into a 2 mL centrifuge tube. Then, 900 μL acetonitrile containing labeled internal standards was added for protein precipitation. The solution was vortexed and then centrifugated (15,000 rpm, 4 °C) for 15 min. Finally, the supernatant was filtered through a 0.22 μm nylon filter into a 2 mL sample bottle before UPLC-Q-Orbitrap HRMS analysis. Chromatograph separation was achieved through a Waters ACQUITY UPLC BEH HILIC column (2.1 mm × 100 mm, 1.7 μm, Waters, Milford, MA, USA). The mobile phase consisted of 95% acetonitrile (v/v, Eluent A) and water (Eluent B); both contained 10 mM ammonium formate. The flow rate was 0.35 mL/min, and the gradient condition was as follows: −2.0 to 0.0 min, 5% B; 0.0–0.5 min, 5% B; 0.5–3.5 min, 5–20% B; 3.5–4.5 min, 20–40% B; 4.5–5.1 min, 40% B; 5.1–5.2 min, 40–5% B. The total method ended in 6 min. Quantification was conducted by a Q Exactive Orbitrap HRMS in positive mode. The average recoveries ranged from 79.1% to 110.0%, with intra- and inter-day relative standard deviation of 1.7–7.8% and 0.6–11.7%, respectively. Details of the modified method are shown in Supplementary Materials (Tables S2 and S3). Furthermore, standards (TMAO and its precursors) in the analysis were purchased from Sigma-Aldrich (Buchs, Switzerland). Isotope-labeled internal standards (IS) including D9-choline, D9-TMAO, D9-L-carnitine, and D11-betaine were obtained from Cambridge Isotope Laboratories, Inc. (Andover, MA, USA).

### 2.4. Statistical Analysis

Urinary TMAO and precursors were ln-transformed to reduce the influence of outliers due to their strong right-skewed distribution. Geometric means (GMs) and 95% confidence intervals (CIs) were used to describe the distributions of urinary TMAO and precursors. Specific gravity (SG) of children’s urine samples was determined to adjust the variability of urinary dilution. The characteristics of the children were described using median and interquartile range (IQR) for continuous variables and frequencies for categorical variables.

Spearman rank correlation coefficients were calculated to reflect the correlation between TMAO and precursors. Generalized linear regression models were conducted to determine the association of specific food consumption and frequency of fish consumption and dietary diversity score with urinary TMAO and precursors concentrations. It was worth nothing that infant food and seasoning intakes were included in the analysis due to the low amount of consumption. Potential confounders were composed of the following variables: child’s sex, age, passive smoking status, total physical activity, taste preference, vitamin intake, and BMI. Potential covariates were confirmed on prior literature and statistical consideration. The covariates were included in the models if they related to TMAO and precursors concentrations, or changed the coefficients of TMAO and precursors concentrations by more than 10%.

All reported probability values were two-tailed, and the criterion for significance was set at *p* < 0.05. Statistical analysis was performed with SAS (version 9.4, SAS Institute Inc., Chicago, IL, USA) and R (version4.1.1, R Core Development Team, Boston, MA, USA).

## 3. Results

### 3.1. Characteristics of the Study Participants

The sample size of this study was 474 children. The characteristics of the children were shown in Table 1. The median age of the children (boy, *n* = 249, girl, *n* = 225) was 118 months (IQR: 117–120). A total of 227 (47.89%) children reported passive smoking and 247 (52.11%) stated no passive smoking. The median BMI was 17.41. The number of children who exercise more than 7 h per week was 171. Most of the children (*n* = 455) had no vitamin usage. The median daily energy intake was 1385.79 kcal. No significant difference was found in socio-demographic characteristics between participants included in the current study (*n* = 474) and those in the initial cohort (*n* = 482, Table S4).

### 3.2. Urinary Concentrations of TMAO and Precursors

The urinary concentrations of TMAO and precursors are illustrated in Table 2. Urinary TMAO and precursors concentrations were detected in all the children. The GM values of unadjusted concentrations of TMAO, TMA, choline, betaine, L-carnitine, and acetyl-L-carnitine were 323.46 μM, 2.29 μM, 31.16 μM, 73.82 μM, 38.85 μM, and 14.92 μM, respectively. The median of SG-adjusted urinary TMAO, TMA, choline, betaine, L-carnitine, and acetyl-L-carnitine were 398.68 μM, 3.01 μM, 42.74 μM, 98.80 μM, 56.05 μM, and 21.53 μM, respectively. In spearman analysis, urinary TMAO was positively correlated with TMA, choline and betaine (r = 0.11 to 0.57, *p* < 0.05) (Figure S1).

### 3.3. Associations of Food Group Intake with TMAO and Precursors Concentrations

**Figure 1:**
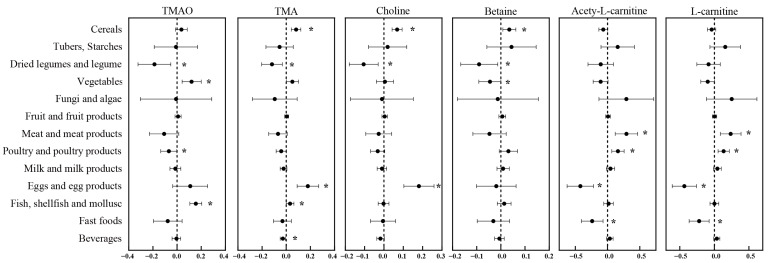
Associations of trimethylamine-N-oxide and its precursors with the food categories from the 24 h dietary recall. Notes: Models were adjusted for sex, age, passive smoking, total physical activity, taste preference, vitamin usage, total energy intake, BMI, and food items. Asterisk means a *p* < 0.05 for the associations of trimethylamine-N-oxide and its precursors with the food categories. The black points and lines mean regression coefficient and 95% CI of the associations, respectively.

The relations of food group intake based on the 24 h dietary recall with urinary TMAO and precursors concentrations were presented in Figure 1 and Table S5. The fish and vegetables intakes were significantly positively associated with urinary TMAO concentration (β = 0.155; β = 0.120, *p* < 0.05). However, dried legumes and poultry intakes were in negative associations with urinary TMAO concentration (β = −0.187; β = −0.068, *p* < 0.05). Meanwhile, fish intake was also significantly negatively related to urinary TMA concentration (β = 0.032, *p* < 0.05). Furthermore, egg intake was significantly positively associated with TMA and choline (β = 0.179; β = 0.181, *p* < 0.05). The significant effects of cereals and dried legumes on precursors were limited to TMA, choline, and betaine. The analysis also identified positive associations between acetyl-L-carnitine and L-carnitine with meat and poultry intake (β = 0.236; β = 0.134, *p* < 0.05), and negative associations with eggs and fast foods intake (β = −0.435; β = −0.221, *p* < 0.05).

**Figure 2:**
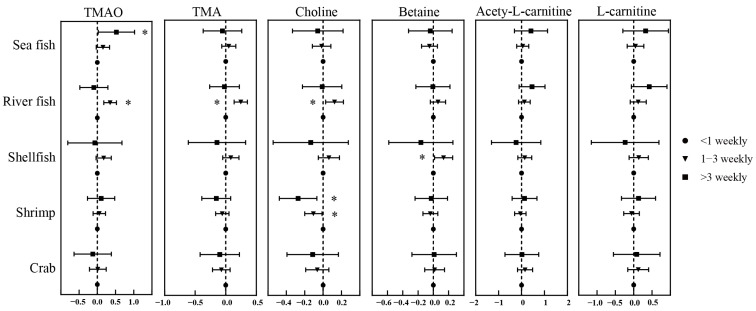
Associations of trimethylamine-N-oxide and its precursors with detailed components of fish from simple FFQ. Notes: Models were adjusted by sex, age, passive smoking, total physical activity, taste preference, vitamin usage, total energy intake BMI, and frequency of eating fish. Asterisk means a *p* < 0.05 for the associations of trimethylamine-N-oxide and its precursors with the frequency of fish consumption. The black points and lines mean regression coefficient and 95%CI of the associations, respectively.

The frequency of fish and aquatic products consumption was also associated with the concentrations of TMAO and precursors (Figure 2 and Table S6). Compared with children who took sea fish less than once a week, children who ate more than three times a week were associated with a coefficient of 0.525 (*p* = 0.039) increases in TMAO concentration. Furthermore, the levels of urinary TMAO, TMA, and choline in children who ate river fish 1–3 times a week were significantly higher (β: 0.125 to 0.351, *p* < 0.05) than in those who ate less. Eating shellfish 1–3 times weekly had a higher (β = 0.134, *p* = 0.027) betaine concentration in urine compared with less than once a week. However, a high frequency of eating shrimp was inversely associated with urinary choline concentration (1–3 weekly: β = −1.106, *p* = 0.027; >3 weekly: β = −0.272, *p* = 0.009).

### 3.4. Associations of Dietary Diversity Scores and TMAO and Precursors Concentrations

Finally, we assessed the association of dietary diversity scores with the concentrations of TMAO and precursors in urine (Table 3). DDS_10_, DDS, and FVS were positively correlated with TMAO concentration after adjusting for potential confounders (DDS_10_: β = 0.091, *p* = 0.008; DDS: β = 0.087, *p* = 0.007; FVS: β = 0.027, *p* = 0.018). DDS_10_ was also related to the level of TMA in urine (β = 0.054, *p* = 0.012). Nevertheless, DDS was inversely associated with the concentrations of betaine, acetyl-L-carnitine, and L-carnitine (betaine: β = −0.036, *p* = 0.045; acetyl-L-carnitine: β = −0.101, *p* = 0.029; L-carnitine: β = −0.085, *p* = 0.032).

## 4. Discussion

In the present study, we found that certain kinds of food components (fish-related food, vegetables, cereals, meats, and poultry) were related to urinary TMAO and precursors, respectively. The habits of eating fish were also associated with TMAO, TMA, choline, and betaine, rather than L-carnitine and acetyl-L-carnitine. A significant increase in urinary TMAO concentration was observed following DDS_10_, DDS, and FVS. To our best knowledge, this is the first study that elucidated the associations of all food components, habits of eating fish, and dietary diversity scores with urinary TMAO and precursors in school-age children.

In the present study, we reported urinary TMAO, TMA, choline, betaine, L-carnitine, and acetyl-L-carnitine levels in school-age children aged ten years, which were of less concern in previous research. Previous studies focused attention on the level of TMAO in the subject with abnormal function of the kidney [12,34], chronic cardiovascular disease [1,3], or received dietary interventions [23,39] (Table S7). A cross-sectional study found that children with CKD G1 had a higher level of urinary TMAO (Median: 271.1 ng/mg Cr) compared to those with CKD G2-G4 (Median: 183.8 ng/mg Cr) [12]. A similar result was presented in another study conducted among 86 children with CKD [40]. Urinary TMAO concentrations in the present study were much higher than that in these studies. Since urine is the predominant excretion pathway of TMAO, kidney function playing an important role might explain the difference [41]. Another study in France elucidated the mean concentration of TMAO in urine samples of healthy subjects was 118.66 μmol/mmol creatinine [42]. The Urine Metabolome Database (UMDB) reported the mean urinary TMAO concentration in adults was 91.0 μmol/mmol creatinine [43]. The geometric mean of the TMAO level in the present study was 44.99 μmol/mmol creatinine (Table S8), which was lower than the results of a study in France and UMDB. However, the level of urinary TMAO among healthy newborns (mean: 12.2 μmol/mmol creatinine) was lower than our results [44]. The better kidney, liver, and gut microbiome function with age increasing could explain the difference among different age groups [35,43,44]. The precursors of TMAO in our study contained similar levels of these metabolomes in UMDB [43].

In our study, we observed positive associations of fish and seafood product intake with urinary TMAO and TMA concentrations. Meanwhile, the frequency of fish eating was also positively related to the level of urinary TMAO and TMA. These findings among 10-year-old children were consistent with several previous studies [23,25,26,28,45,46]. A study conducted among 4680 adults in Japan reported urinary TMAO was directly associated with fish intake, and the correlations were stronger compared with the Western population [25]. For instance, a null association was shown in the study of Norwegians [47]. Differences in dietary habits and ethnicity may account for discordance in observations. Another study conducted among 620 male participants in America found that TMAO concentration was significantly associated with fish intake [48]. Moreover, the consumption of fish food also showed high levels of TMAO in plasma and urine in Germany [16]. Associations across fish intake might be attributed to the fact that fish and other seafood were rich in free TMAO [49]. Thus, TMAO is discussed as a marker for current fish consumption; although, fish is not the only dietary source [50]. On the other hand, fish was conventionally regarded as rich in long-chain omega-3 polyunsaturated fatty acids (PUFAs) [28], which were reported to be related to a decreased risk of diseases such as cancer and heart disease [25]. In terms of both, the effect of fish intake needs further discussion.

Meat and poultry are rich sources of carnitine, and meat has a 5 to 20 times higher carnitine content compared to poultry [51,52]. In our study, consumption of meat and poultry was associated with L-carnitine and acetyl-L-carnitine rather than TMAO in urine. These are in line with a study in German that association with meat consumption was found for urinary carnitine, not for urinary TMAO [16]. Two additional German studies showed relations between meat intake and plasma TMAO [21,53]. However, red meat consumption was not significantly related to urinary TMAO in two other studies [26,54]. Remarkably, meat intake had a stronger association with urinary TMAO than plasma TMAO. Moreover, although TMAO-precursors TMA could be produced by carnitine, the conversion was affected by gut microbes [21]. The difference in their gut microbiota composition in populations would be also associated with TMAO and carnitine levels.

Unlike meat consumption, egg intake was inversely related to carnitine in the present study. A possible explanation could be that eggs were a substitute for meat in a day’s food intake. Nevertheless, eggs had a positive association with urinary TMA and choline, which was attributed to the fact that eggs are rich in choline. Dairy, another choline-rich food item, was not found to relate to TMAO and precursors in the present study. However, two studies conducted in adults showed the associations of dairy intake with plasma TMAO concentration [33,55]. A low dairy intake of children in our study might explore the difference. As for plant-based foods, most of the related studies found no correlations between TMAO and ingestion of fruits, vegetables [45], and cereals [56]. An inverse result was observed whereby some plant-based foods (cereals, legumes, and vegetables) were related to urinary TMAO and precursors. Although plant-based food did not have a direct association with TMAO, there was a distinct gut microbial profile in individuals with plant-based diets compared with animal-related diets [57].

Our results from the analyses of the dietary diversity scores showed positive associations with the concentration of urinary TMAO. In turn, we found an inverse relationship between urinary carnitine and dietary diversity score. The findings indicated the opposite because dietary diversity scores are a consequence of the positive association with fish as opposed to negative influences of meat intake [37]. The consumption of fish might lead to the intake of direct TMAO. Although the diversity of the diet was associated with more fiber intake, fiber might reshape intestinal microbiota composition and then affect TMAO generation. This speculative hypothesis was not indicated in our study but rather supported by a 30-day high-fiber diet intervention study on children with obesity [18].

Our study has several strengths and limitations. To our knowledge, this is the first epidemiological study evaluating associations of diet composition and diversity scores with TMAO and its precursors simultaneously. The subjects of this study were school-age free-living children rather than adults with specific diseases or dietary modulation in previous research; although, the sample size of this study was limited. Nevertheless, our 24 h dietary recall data from one day have their own sources of error; although, we have chosen children eating their usual normal diet. Moreover, we found analogous results from both the 24 h dietary recall and eating fish habits, which provide strong internal validation. Another limitation was the cross-sectional design, which limited the inference of causality between diet and TMAO and its precursors. As with all observational studies, we cannot entirely consider the possibility of unmeasured or unknown confounding factors that might bias the findings in this study, such as kidney function. However, the assessment of general characteristics and calibration of urine specific gravity minimized potential confounding. Furthermore, due to the lack of blood samples, we did not reflect on the direct influence of diet on TMAO concentration. However, previous literature [30] has reported a strong correlation between urinary and plasma TMAO, and the urinary TMAO concentration was a noninvasive biomarker appropriate for free-living children. Further study is warranted to confirm whether repeated dietary data are associated with TMAO and its precursors in urine and blood samples.

## 5. Conclusions

Among free-living individuals, especially school-age children, urinary TMAO and its precursors might be affected by certain food compositions, such as fish, meat, and eggs. A high level of dietary diversity had a positive association with TMAO concentration. Prospective studies are needed to elucidate the relationships between diet, gut microbiome, TMAO, and the growth of children.

## Figures and Tables

**Table 1:** Sociodemographic characteristics of the study participants (*n* = 474).

Characteristics	*N* (%)/Median (IQR)
Sex	
Boy	249 (52.53)
Girl	225 (47.47)
Age (month)	118 (117, 120)
BMI	17.41 (15.69, 20.59)
Passive smoking	
Yes	227 (47.89)
No	247 (52.11)
Total physical activity	
≤3 h weekly	169 (35.65)
3–7 h weekly	134 (28.27)
>7 h weekly	171 (36.08)
Vitamin usage	
Yes	19 (4.01)
No	455 (95.99)
Taste preferences	
Lightly flavor	329 (69.41)
Soft oily and salty	117 (24.68)
Severe oily and salty	28 (5.91)
Daily energy intake (kcal)	1385.79 (1078.33, 1868.42)

IQR: interquartile range; BMI: body mass index.

**Table 2:** The urinary concentration of trimethylamine-N-oxide and its precursors in school-age children.

Analytes	GM (95% CI)	Quantile Distribution				
		P5	P25	P50	P75	P95
Unadjusted (μM)						
TMAO	323.46 (292.38, 357.85)	52.29	156.63	324.75	616.25	2254.80
TMA	2.29 (2.13, 2.46)	0.54	1.29	2.62	3.97	7.09
Choline	31.16 (29.01, 33.45)	7.32	19.78	34.90	57.01	88.40
Betaine	73.82 (68.52, 79.52)	16.29	43.97	85.88	132.10	229.41
L-carnitine	38.85 (34.31, 44.00)	2.70	14.07	41.02	108.10	344.78
Acetyl-L-carnitine	14.92 (12.92, 17.24)	0.90	4.78	15.75	48.57	204.20
SG-adjusted (μM)						
TMAO	458.04 (421.88, 497.31)	132.53	262.53	398.68	661.14	3016.51
TMA	3.23 (3.07, 3.40)	1.41	2.24	3.01	4.22	8.89
Choline	44.01 (42.08, 46.04)	21.88	31.74	42.74	57.01	97.89
Betaine	104.33 (99.60, 109.27)	51.28	75.95	98.802	133.70	225.37
L-carnitine	54.80 (49.32, 60.89)	7.89	22.50	56.05	126.79	306.60
Acetyl-L-carnitine	21.05 (18.64, 23.78)	2.11	7.48	21.53	56.36	165.38

GM: Geometric mean; CI: confidence interval; SG: Specific gravity.

**Table 3:** Association of trimethylamine-N-oxide and its precursors with indicators of dietary diversity.

	DDS_10_		DDS		FVS	
	β (95% CI)	*p*	β (95%CI)	*p*	β (95% CI)	*p*
TMAO	0.091 (0.024, 0.158)	0.008	0.087 (0.024, 0.149)	0.007	0.027 (0.005, 0.049)	0.018
TMA	0.054 (0.012, 0.097)	0.012	0.036 (−0.004, 0.076)	0.076	0.009 (−0.005, 0.023)	0.214
Choline	0.024 (−0.013, 0.061)	0.199	0.013 (−0.022, 0.048)	0.466	0.002 (−0.011, 0.014)	0.790
Betaine	−0.029 (−0.068, 0.009)	0.133	−0.036 (−0.072, −0.001)	0.045	−0.008 (−0.021, 0.005)	0.214
Acetyl-L-carnitine	−0.093 (−0.190, 0.005)	0.062	−0.101 (−0.192, −0.010)	0.029	−0.020 (−0.052, 0.012)	0.218
L-carnitine	−0.083 (−0.167, 0.0004)	0.051	−0.085 (−0.164, −0.007)	0.032	−0.014 (−0.041, 0.014)	0.336

Models were adjusted for sex, age, passive smoking, total physical activity, taste preference, vitamin usage, total energy intake BMI, and indicators of dietary diversity.

## Data Availability

The data presented in this study are available on request from the corresponding author. The data are not publicly available due to privacy.

## Supplementary Materials

The following are available online at https://www.mdpi.com/article/10.3390/nu14163419/s1, Figure S1: Correlations of TMAO and its precursors in urine. Notes: The values in this figure represented the Pearson correlation coefficients. Table S1: Ten food groups of dietary diversity scores. Table S2: Levels of calibration curves for Trimethylamine N-Oxide (TMAO) and its precursors. Table S3: Intra-day and inter-day assay precision and accuracy for urine sample. Table S4: Comparisons of Characteristics between total sample (*n* = 482) and included children (*n* = 474). Table S5: Association of trimethylamine-N-oxide and its precursors with the food categories from the 24 h recall data. Table S6: Association of trimethylamine-N-oxide and its precursors with detailed components of fish from simple FFQ. Table S7: Comparison of Trimethylamine-N-oxide (TMAO) concentrations in previous literature. Table S8: The urinary concentrations of trimethylamine-N-oxide and its precursors in school-age children.
