# Supplementary material for: Associations of Diet with Urinary Trimethylamine-N-Oxide (TMAO) and Its Precursors among Free-Living 10-Year-Old Children: Data from SMBCS
Source: Nutrients. 2022 Aug 19;14(16):3419. doi: 10.3390/nu14163419 (PMC9413070; doi:10.3390/nu14163419)
Supplement: Supplementary file 1 [file nutrients-14-03419-s001.zip › nutrients-1863010-supplementary.pdf]

Table S1. Ten food groups of dietary diversity scores.

**Food Groups**	**Foods**
1. Cereals, roots and tubers	rice, wheat, maize, cassava, potatoes
2. Vitamin A rich fruits and vegetables	acacia leaves, amaranth, sweet red pepper, Chinese cabbage, carrot, cashew leaf, cassava leaf, chrysanthemum leaf, cowpea leaf, fennel, mustard greens, mango, papaya, etc.
3. Other fruits	apple, banana, orange, pear
4. Other vegetables	cabbage, green beans, broccoli, mushrooms, cucumber, eggplant, cauliflower
5. Legumes, pulses and nuts	beans, peas, peanuts, soy bean
6. Oils and Fats	vegetable oil, lard, butter, ghee
7. Meat, poultry, fish	beef, pork, chicken, turkey, fish, shellfish
8. Dairy	milk, yogurt, cheese, ice cream
9. Eggs	
10. Other food	sweets, chips, soda, condiments, etc.

Table S2. Levels of calibration curves for urinary Trimethylamine N-Oxide (TMAO) and its precursors.

Analytes	Level 0	Level 1	Level 2	Level 3	Level 4	Level 5	Level 6	Level 7
TMA	0	10	25	50	100	200	500	1000
Choline	0	200	500	1000	2000	4000	10000	20,000
Betaine	0	200	500	1000	2000	4000	10000	20,000
Acetyl-L-carnitine	0	200	500	1000	2000	4000	10000	20,000
L-carnitine	0	200	500	1000	2000	4000	10000	20,000
TMAO	0	1000	2500	5000	10,000	20,000	50,000	100,000

Table S3. Intra-day and inter-day assay precision and accuracy for urine sample.

Analyte	Sample	Added (μg/L)	Intra-day Mean (μg/L)	Intra-day CV (%)	Intra-day Recovery (%)	Inter-day Mean (μg/L)	Inter-day CV (%)	Inter-day Recovery (%)
TMA	Blank	0.0	4.5	-	-	6.2	-	-
	QC-L	25.0	30.2	4.6	103.0	32.3	6.3	104.2
	QC-M	100.0	98.7	2.4	94.2	11.4	2.2	95.1
	QC-H	500.0	454.6	2.8	90.0	465.7	2.7	91.9
Choline	Blank	0.0	65.3	-	-	58.6	-	-
	QC-L	500.0	587.8	2.5	104.5	592.2	0.8	106.7
	QC-M	2000.0	2107.6	2.1	102.1	2113.9	1.3	102.7
	QC-H	10000.0	9162.8	2.3	91.0	9274.0	1.5	92.2
Betaine	Blank	0.0	118.9	-	-	115.4	-	-
	QC-L	500.0	615.9	1.7	99.4	631.3	3.5	103.2
	QC-M	2000.0	2069.4	3.8	97.5	2132.6	3.6	100.8
	QC-H	10000.0	8680.2	2.9	85.6	9.4	3.8	88.8
Acetyl-L-Carnitine	Blank	0.0	7.3	-	-	19.0	-	-
	QC-L	500.0	489.6	5.1	96.4	478.3	2.4	91.9
	QC-M	2000.0	1770.1	5.9	88.1	1719.2	2.6	85.0
	QC-H	10000.0	7918.6	4.4	79.1	7661.4	3.0	76.4
L-Carnitine	Blank	0.0	120.3	-	-	112.2	-	-
	QC-L	500.0	670.5	2.7	110.0	697.3	4.5	117.1
	QC-M	2000.0	2309.7	5.0	109.5	245.5	4.7	114.7
	QC-H	10000.0	9758.9	2.3	96.4	989.3	3.2	97.8

TMAO	Blank	0.0	382.7	-	-	376.2	-	-
	QC-L	2500.0	3131.7	4.0	110.0	3168.3	1.2	111.7
	QC-M	10000.0	11312.5	3.2	109.3	11254.6	2.6	108.8
	QC-H	50000.0	45033.0	1.7	89.3	45257.2	0.9	89.8

Note: Blank: blank urine sample; QC-L: low level quality control sample; QC-M: medium level quality control sample; QC-H: high level quality control sample

Table S4. Comparisons of Characteristics between total sample (n = 482) and included children (n = 474).

Characteristics	Total children (n = 482)	Included children (n = 474)	p-value
Sex			
Boy	254 (52.70)	249 (52.53)	0.959
Girl	228 (47.30)	225 (47.47)	
Age (month)	118 (117, 120)	118 (117, 120)	
BMI (kg/m^2)	17.44 (15.72, 20.70)	17.41 (15.69, 20.59)	
Passive smoking			
Yes	233 (48.34)	227 (47.89)	0.889
No	249 (51.66)	247 (52.11)	
Total physical activity			
≤3 h weekly	173 (35.89)	169 (35.65)	0.995
3-7 h weekly	135 (28.01)	134 (28.27)	
>7 h weekly	174 (36.10)	171 (36.08)	
Vitamin usage			
Yes	19 (3.94)	19 (4.01)	0.958
No	463 (96.06)	455 (95.99)	
Taste preferences			
Lightly flavor	332 (68.88)	329 (69.41)	0.984
Soft oily and salty	121 (25.10)	117 (24.68)	
Severe oily and salty	29 (6.02)	28 (5.91)	
Daily energy intake (kcal)	1380.78 (1060.36, 1868.42)	1385.79 (1078.33, 1868.42)	

IQR: interquartile range; BMI: body mass index. p-value was tested by Chi-square test.

Table S5. Association of trimethylamine-N-oxide and its precursors with the food categories from the 24-hr recall data.

	TMAO	TMA	Choline	Betaine	Acetyl-l-carnitine	L-carnitine
	β (95%CI)	β (95%CI)	β (95%CI)	β (95%CI)	β (95%CI)	β (95%CI)
Cereals and Cereal products	0.036 (−0.013, 0.084)	0.082 (0.042, 0.121)*	0.068 (0.042, 0.094)*	0.035 (0.008, 0.062)*	−0.066 (−0.135, 0.004)	−0.039 (−0.099, 0.021)
Tubers, Starches and products	−0.009 (−0.189, 0.171)	−0.055 (−0.168, 0.058)	0.019 (−0.080, 0.118)	0.044 (−0.058, 0.146)	0.157 (−0.102, 0.416)	0.158 (−0.064, 0.381)
Dried legumes and legume products	−0.187 (−0.323, −0.052)*	−0.118 (−0.203, −0.032)	−0.105 (−0.179, −0.030)*	−0.090 (−0.166, −0.013)*	−0.106 (−0.303, 0.091)	−0.084 (−0.254, 0.085)
Vegetables and vegetable products	0.120 (0.041, 0.200)*	0.051 (0.001, 0.101)*	0.005 (−0.039, 0.049)	−0.045 (−0.091, −0.000)*	−0.107 (−0.223, 0.008)	−0.096 (−0.196, 0.003)
Fungi and algae	−0.008 (−0.304, 0.287)	−0.095 (−0.280, 0.091)	−0.010 (−0.173, 0.152)	−0.013 (−0.180, 0.155)	0.292 (−0.133, 0.717)	0.251 (−0.115, 0.616)
Fruit and fruit products	0.008 (−0.017, 0.033)	0.004 (−0.012, 0.020)	0.004 (−0.010, 0.018)	0.006 (−0.008, 0.020)	0.008 (−0.028, 0.044)	0.002 (−0.029, 0.033)
Nuts and seeds	0.147 (−0.345, 0.638)	−0.068 (−0.377, 0.241)	−0.075 (−0.345, 0.196)	0.035 (−0.243, 0.313)	−0.621 (−1.327, 0.086)	−0.500 (−1.108, 0.109)
Meat and meat products	−0.107 (−0.228, 0.015)	−0.068 (−0.145, 0.008)	−0.027 (−0.094, 0.040)	−0.047 (−0.116, 0.022)	0.293 (0.119, 0.467)*	0.236 (0.086, 0.386)*
Poultry and poultry products	−0.068 (−0.135, −0.002)*	−0.041 (−0.083, 0.001)	−0.032 (−0.069, 0.004)	0.031 (−0.007, 0.069)	0.161 (0.066, 0.256)*	0.134 (0.053, 0.216)*
Milk and milk products	−0.014 (−0.059, 0.031)	−0.022 (−0.050, 0.006)	−0.010 (−0.035, 0.014)	0.009 (−0.016, 0.035)	0.046 (−0.019, 0.111)	0.044 (−0.013, 0.100)
Eggs and egg products	0.109 (−0.036, 0.253)	0.179 (0.090, 0.269)*	0.181 (0.103, 0.259)*	−0.019 (−0.101, 0.063)	−0.422 (−0.627, −0.217)*	−0.435 (−0.610, −0.260)*
Fish, shellfish and mollusc	0.155 (0.106, 0.204)*	0.032 (0.0001, 0.064)*	−0.002 (−0.030, 0.026)	0.014 (−0.015, 0.042)	0.016 (−0.057, 0.089)	0.001 (−0.063, 0.064)
Infant foods	4.495 (−4.569, 13.558)	2.728 (−2.978, 8.433)	1.352 (−3.638, 6.341)	−0.813 (−5.953, 4.327)	−6.512 (−19.584, 6.559)	−6.431 (−17.678, 4.817)
Snacks, cookies	0.109 (−0.086, 0.303)	0.075 (−0.047, 0.197)	0.007 (−0.100, 0.114)	−0.109 (−0.219, 0.001)	−0.177 (−0.457, 0.103)	−0.141 (−0.382, 0.100)
Fast foods	−0.077 (−0.195, 0.041)	−0.031 (−0.105, 0.043)	−0.005 (−0.070, 0.060)	−0.031 (−0.098, 0.036)	−0.239 (−0.407, −0.070)*	−0.221 (−0.366, −0.076)*
Beverages	−0.005 (−0.041, 0.030)	−0.028 (−0.050, −0.006)*	−0.018 (−0.037, 0.002)	−0.006 (−0.026, 0.014)	0.036 (−0.015, 0.087)	0.036 (−0.008, 0.080)
Alcohol	−0.022 (−0.281, 0.237)	−0.051 (−0.214, 0.112)	−0.054 (−0.197, 0.088)	0.032 (−0.115, 0.179)	0.234 (−0.140, 0.607)	0.155 (−0.166, 0.477)
Sugar, honey	−0.061 (−0.390, 0.269)	0.026 (−0.181, 0.233)	−0.055 (−0.236, 0.126)	−0.058 (−0.245, 0.128)	0.015 (−0.460, 0.490)	0.124 (−0.284, 0.532)
Dressing	−0.209 (−0.888, 0.470)	−0.206 (−0.633, 0.221)	−0.143 (−0.516, 0.231)	0.130 (−2.255, 0.515)	−0.259 (−1.239, 0.721)	−0.176 (−1.019, 0.667)

Data were adjusted for sex, age, passive smoking, total physical activity, taste preference, vitamin usage, total energy intake, BMI and food items.

Asterisk means a $p < 0.05$ for the associations of trimethylamine-N-oxide and its precursors with the food categories.

Table S6. Association of trimethylamine-N-oxide and its precursors with detailed components of fish from simple FFQ.

	TMAO		TMA		Choline		Betaine		Acetyl-l-carnitine		L-carnitine	
	β (95%CI)	*p*	β (95%CI)	*p*	β (95%CI)	*p*	β (95%CI)	*p*	β (95%CI)	*P*	β (95%CI)	*p*
Sea fish												
< 1 weekly	Ref.		Ref.		Ref.		Ref.		Ref.		Ref.	
1−3 weekly	0.155 (−0.03, 0.336)	0.095	0.050 (−0.065, 0.165)	0.392	−0.016 (−0.116, 0.085)	0.762	−0.051 (−0.154, 0.053)	0.338	0.052 (−0.211, 0.316)	0.697	0.049 (−0.178, 0.276)	0.672
>3 weekly	0.525 (0.027, 1.024)	0.039	−0.053 (−0.369, 0.263)	0.742	−0.058 (−0.334, 0.218)	0.679	−0.039 (−0.324, 0.245)	0.786	0.415 (−0.308, 1.138)	0.260	0.326 (−0.296, 0.949)	0.304
River fish												
< 1 weekly	Ref.		Ref.		Ref.		Ref.		Ref.		Ref.	
1−3 weekly	0.351 (0.178, 0.524)	<0.001	0.246 (0.137, 0.354)	<0.001	0.125 (0.028, 0.221)	0.011	0.061 (−0.039, 0.160)	0.234	0.124 (−0.129, 0.378)	0.336	0.123 (−0.095, 0.341)	0.268
>3 weekly	−0.095 (−0.480, 0.291)	0.631	−0.020 (−0.262, 0.222)	0.871	−0.009 (−0.224, 0.205)	0.932	−0.006 (−0.228, 0.217)	0.962	0.460 (−0.105, 1.025)	0.111	0.423 (−0.063, 0.909)	0.088
Shellfish												
< 1 weekly	Ref.		Ref.		Ref.		Ref.		Ref.		Ref.	
1−3 weekly	0.174 (−0.036, 0.384)	0.105	0.083 (−0.049, 0.216)	0.218	0.064 (−0.052, 0.179)	0.281	0.134 (0.015, 0.253)	0.027	0.143 (−0.161, 0.446)	0.356	0.138 (−0.123, 0.399)	0.300
>3 weekly	−0.066 (−0.810, 0.678)	0.862	−0.143 (−0.611, 0.326)	0.550	−0.134 (−0.544, 0.275)	0.520	−0.163 (−0.583, 0.257)	0.447	−0.236 (−1.311, 0.839)	0.667	−0.233 (−1.158, 0.69)	0.621
Shrimp												
< 1 weekly	Ref.		Ref.		Ref.		Ref.		Ref.		Ref.	
1−3 weekly	0.054 (−0.118, 0.226)	0.540	−0.057 (−0.165, 0.052)	0.305	−0.106 (−0.200, −0.012)	0.027	−0.039 (−0.136, 0.059)	0.437	−0.049 (−0.297, 0.199)	0.699	−0.059 (−0.273, 0.155)	0.589
>3 weekly	0.105 (−0.270, 0.480)	0.582	−0.154 (−0.390, 0.081)	0.200	−0.272 (−0.477, −0.068)	0.009	−0.026 (−0.239, 0.186)	0.809	0.134 (−0.407, 0.675)	0.627	0.132 (−0.333, 0.597)	0.579
Crab												
< 1 weekly	Ref.		Ref.		Ref.		Ref.		Ref.		Ref.	
1−3 weekly	0.013 (−0.216, 0.243)	0.909	−0.072 (−0.216, 0.072)	0.328	−0.063 (−0.189, 0.063)	0.328	0.017 (−0.113, 0.146)	0.804	0.153 (−0.177, 0.484)	0.363	0.124 (−0.160, 0.408)	0.392
>3 weekly	−0.126 (−0.638, 0.386)	0.629	−0.097 (−0.419, 0.224)	0.553	−0.113 (−0.394, 0.168)	0.432	0.011 (−0.279, 0.301)	0.943	0.017 (−0.721, 0.754)	0.964	0.083 (−0.552, 0.718)	0.799

Data were adjusted for sex, age, passive smoking, total physical activity, taste preference, vitamin usage, total energy intake BMI and frequency of eating fish.

Table S7. Comparison of Trimethylamine-N-oxide (TMAO) concentrations in previous literature.

Reference	Location	Participants	Sample size	Matrix	Concentration	Unit
Presents study	Jiangsu, China	10-year-old children	474	Urine	323.46 (292.38, 357.85)	µM
					458.04 (421.88, 497.31)	µM (specific gravity adjusted)
					44.99 (41.68, 49.01)	µmol/mmol creatinine
(HSU, C, et al., 2020) [12]	Taiwan, China	Children with CKD G1	79	Urine	271.1 (167.3–417.8)	ng/mg Cr
	Taiwan, China	Children with CKD G2-G4	36	Urine	183.8 (107.5–291.6)	ng/mg Cr
(HSU, C, et al., 2018) [40]	Taiwan, China	Children with CKD G1	60	Urine	344 (200–853)	ng/mg Cr
	Taiwan, China	Children with CKD G2-G3	26	Urine	209 (155–412)	ng/mg Cr
(MASCHKE, S, et al., 1997) [42]	France	Healthy subject		Urine	118.66 ± 83.18	µmol/mmol creatinine
(BOUATRA, S, et al., 2013) [43]	Canada	Adults	22	Urine	91.0	µmol/mmol creatinine
(LÓPEZ-HERNÁNDEZ, Y, et al., 2020) [44]	Mexico	Newborns		Urine	12.2	µmol/mmol creatinine

Table S8. The urinary concentrations of trimethylamine-N-oxide and its precursors in school-age children.

		Quantile distribution				
	GM (95%CI)	P5	P25	P50	P75	P95
Unadjusted (μM)						
TMAO	323.46 (292.38, 357.85)	52.29	156.63	324.75	616.25	2254.80
TMA	2.29 (2.13, 2.46)	0.54	1.29	2.62	3.97	7.09
Choline	31.16 (29.01, 33.45)	7.32	19.78	34.90	57.01	88.40
Betaine	73.82 (68.52, 79.52)	16.29	43.97	85.88	132.10	229.41
L-carnitine	38.85 (34.31, 44.00)	2.70	14.07	41.02	108.10	344.78
Acetyl-l-carnitine	14.92 (12.92, 17.24)	0.90	4.78	15.75	48.57	204.20
Creatintine adjusted (μmol/mM Cre)						
TMAO	44.99 (41.68, 49.01)	12.73	24.91	39.64	68.4	268.49
TMA	0.32 (0.30, 0.34)	0.14	0.22	0.30	0.42	0.85
Choline	4.33 (4.14, 4.54)	2.05	3.10	4.10	5.66	10.49
Betaine	10.27 (9.74, 10.83)	4.70	7.15	10.1	13.73	24.53
L-carnitine	5.40 (4.90, 5.99)	0.77	2.14	6.17	13.10	32.67
Acetyl-l-carnitine	2.08 (1.85, 2.33)	0.20	0.71	2.22	5.95	16.82

GM: Geometric mean; Cre: Creatintine.

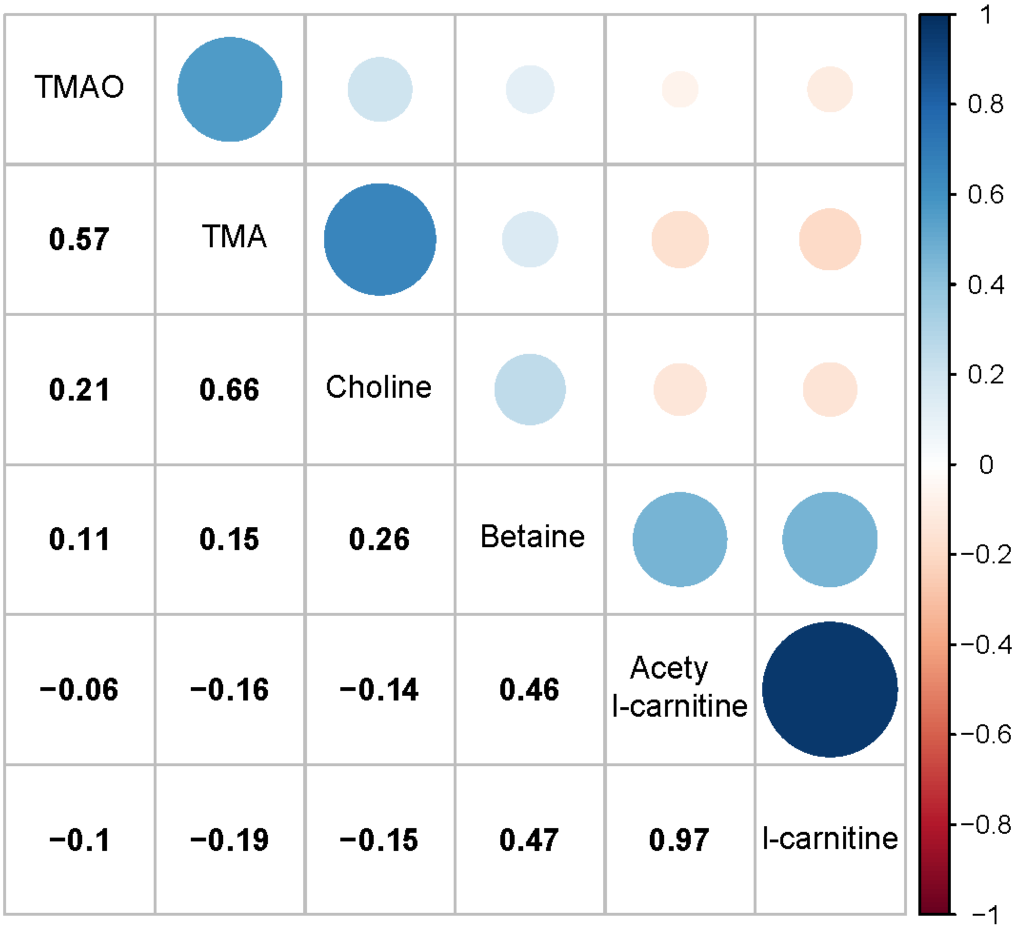

Figure S1. Correlations of TMAO and its precursors in urine. Notes: The values in this figure represented the Pearson correlation coefficients.
